# ISOMAP and machine learning algorithms for the construction of embedded functional connectivity networks of anatomically separated brain regions from resting state fMRI data of patients with Schizophrenia

**DOI:** 10.3934/Neuroscience.2021016

**Published:** 2021-02-19

**Authors:** Ioannis K Gallos, Kostakis Gkiatis, George K Matsopoulos, Constantinos Siettos

**Affiliations:** 1 School of Applied Mathematical and Physical Sciences, National Technical University of Athens, Greece; 2 School of Electrical and Computer Engineering, National Technical University of Athens, Greece; 3 Dipartimento di Matematica e Applicazioni “Renato Caccioppoli”, Università degli Studi di Napoli Federico II, Italy

**Keywords:** resting state fMRI, Schizophrenia, functional connectivity networks, numerical analysis, manifold learning, ISOMAP, feature selection, LASSO, random forests, machine learning

## Abstract

We construct Functional Connectivity Networks (FCN) from resting state fMRI (rsfMRI) recordings towards the classification of brain activity between healthy and schizophrenic subjects using a publicly available dataset (the COBRE dataset) of 145 subjects (74 healthy controls and 71 schizophrenic subjects). First, we match the anatomy of the brain of each individual to the Desikan-Killiany brain atlas. Then, we use the conventional approach of correlating the parcellated time series to construct FCN and ISOMAP, a nonlinear manifold learning algorithm to produce low-dimensional embeddings of the correlation matrices. For the classification analysis, we computed five key local graph-theoretic measures of the FCN and used the LASSO and Random Forest (RF) algorithms for feature selection. For the classification we used standard linear Support Vector Machines. The classification performance is tested by a double cross-validation scheme (consisting of an outer and an inner loop of “Leave one out” cross-validation (LOOCV)). The standard cross-correlation methodology produced a classification rate of 73.1%, while ISOMAP resulted in 79.3%, thus providing a simpler model with a smaller number of features as chosen from LASSO and RF, namely the participation coefficient of the right thalamus and the strength of the right lingual gyrus.

## 1 Introduction

Schizophrenia is a functional chronic mental disorder which affects how a person thinks, feels and acts. Symptoms may vary across patients and they include hallucinations, delusions, thought and movement disorders, disruptions in emotions and cognitive deficits. Prevalence of schizophrenia has reported to be around the 1% of the worldwide population without any significant difference between males and females [1]. In terms of brain connectivity, the “dysconnection hypothesis” has been the dominant theory on the cause of underlying brain malfunction in schizophrenia, suggesting both anatomical and functional brain dysfunction at different levels ranging from neurochemical to emerging functional connectivity impairments, thus demonstrating that schizophrenia could be perceived as a disorder of the human connectome [2]–[5].

Regarding the investigation of the functional brain connectome and neurological disorders, resting state magnetic resonance imaging (rsfMRI) has been proven a powerful tool [6]–[8]. This technique also holds the advantage of being non-invasive and at the same time capable of capturing deep structures of the brain (e.g. sub-cortical regions of the brain, such as the thalamus). Over the years, meta analysis of rsfMRI made possible the identification of the so-called resting state networks (RSN). These networks are reproducible across individual subjects and connect regions of the brain with certain functional properties [9]–[11]. Moreover, rsfMRI has also aided in both the detection of functional connectivity differences and the diagnosis of several neurological disorders, including schizophrenia [4], [12]–[14].

Until today, many studies have proposed several classification/diagnostic biomarkers of schizophrenia [15]–[19]. A typical pipeline for the construction of FCN includes a standard preprocessing routine (e.g. motion correction, removal of non-brain tissue etc.), the use of a parcellation scheme for signal extraction using e.g. Independent Component Analysis (ICA), or the extraction of time series directly in accordance to a predetermined anatomical or functional atlas, and finally the construction of FCN by cross-correlating the corresponding time series. Based on the constructed FCN, specific features are calculated and classifiers are implemented to classify between patients with schizophrenia and healthy controls. Typically, studies suggest the most informative features as biomarkers associated with the disease.

For example, for the construction of FCN and their classification, Cheng et al. [18] used correlation of rsfMRI time series from 278 anatomical atlas-derived nodes from a data set of 29 healthy and 19 schizophrenic subjects. Four different feature sets have been tested, namely, the betweenness centrality scores of all nodes, their ranks, the top ten highest scores and the top ten ranks. The best results were obtained using the rank of betweenness centrality of the top ten hubs of the network. They reported an accuracy of 79% using a single leave one-out cross-validation (LOOCV) and linear standard SVM (LSVM). Xiang et al. [15] also used correlation of time series extracted in accordance with a 246 node anatomical atlas from the COBRE dataset. They computed similar local graph measures to the ones computed here, namely, the degree of node, the participation coefficient, the local clustering coefficient, the betweenness centrality and the local efficiency. The authors utilized different feature selection algorithms such as the Least Absolute Shrinkage Operator (LASSO), Group LASSO (GLASSO) and Sparse Group LASSO (SGLASSO). Using LASSO and 123 features, they obtained a 83.4% classification, a 86.2% with GLASSO and 225 features, and a 93.1% with SGLASSO and 55 features. The evaluation method was one single LOOCV while the parameter tuning for each feature selection algorithm was done via grid search inside a single LOOCV. The classification algorithm that produced the best results in their study was the LSVM. Apparently, the authors, with SGLASSO, which is a two parameter feature selection method, optimized two parameters and trained the LSVM all within a single LOOCV scheme.

Eventually, as stated in [19], the validation method ( cross-validation) affects both the estimation of accuracy and the identification of the dominant features that could be represented as biomarkers. Indeed, Moghimi et al. [19] compared the results of single and double cross-validation schemes to a large dataset comprised of 170 subjects finding that a double cross-validation may lead to even a 20% decrease in the classification performance. The authors assessed more than 19000 global and local network measures and used the Sequential Feature Selection (SFS) algorithm in order to find the most predominant features, that could potentially serve as biomarkers. While using a single cross-validation, their best reported accuracy was 87% with 14 features, following a double cross-validation scheme their best reported accuracy was 73%, resulting from only a single feature. The addition of any other feature decreased the accuracy rate. They finally suggested that the cross-validation procedure may end up with inflated classification rates as a result of over-fitting to a specific dataset. The classification performance using single cross-validation analysis may be also apparently increased by utilizing more features that are less likely to generalize to independent datasets [19], [20].

In a recent study of Cai et al. [21], the authors applied a promising classification framework proposed in [22] to two independent datasets evaluating the within and between site generalizability of the model. The scope of the study was to test the generalizability of the current models for diagnosis and classification of schizophrenia. While with the proposed methodology, Du et al. [22] reported a 93% accuracy rate, the authors in [21] found a within-site accuracy rate of 73% and between-site accuracy of 70%. Finally, Cai et al. [21] attributed the findings to overfitting, a possible heterogeneity of their validating datasets and possible presence of noise, even if a comprehensive preprocessing routine had been applied. Interestingly, the approximately 20% difference in the expected accuracy was found also in the study of Moghimi et al. [19] between single and double cross-validation schemes.

Therefore, despite the fact that in some studies the reported classification performance is relatively high [15], [22], in general the obtained proposed accuracy rates and suggested biomarkers are diverse [19]–[21], [23]. There are several reasons why this happens. First of all, a single cross-validation procedure may end up with an over-optimistic estimate of model's performance [20], [24] especially when feature selection takes place [19]. Another reason is the small sample size [25], [26]. Also the pre-processing procedure may play an important role to the performance of the final classification model [19]. Thus, finding reliable/reproducible biomarkers (e.g. that generalize among different individuals) for schizophrenia remains a challenging problem [19], [21], [23], [25].

Besides the use of linear correlation for the construction of FCN, nonlinear manifold learning algorithms such as Isometric Mapping (ISOMAP) and Diffusion Maps, have been also applied for the construction of FCN [16], [27]. Other approaches such as cross-recurrence analysis and multilayer modelling [28] have been also proposed. Anderson and Cohen [16] used ISOMAP for the construction of embedded low-dimensional FCN for the classification between controls and schizophrenia patients using the COBRE dataset. ROIs were acquired using single subject ICA (for a review on ICA see in [29]). The analysis revealed differences in small-world properties among groups and 13 global-graph theoretic features led to a reported 65% accuracy rate (evaluating the model using a 10-fold cross validation scheme). Gallos et al. [27] used both linear (multidimensional scaling) and nonlinear manifold approaches (ISOMAP, Locally Linear Embedding, kernel PCA and Diffusion Maps) to construct embedded FCN based on the COBRE dataset. The classification performance of the global network properties was tested using key global graph-theoretic properties and several machine learning techniques including radial SVM and neural networks. The performance of two widely used metrics for the construction of FCN metrics, namely the Euclidean distance and the cross correlation metric was also assessed. As in [16] the analysis was performed based on the global graph theoretical measures.

Thus, here, building on recent methodological advances [16], [27], [30], we used ISOMAP to construct embedded FCN towards the classification of 71 schizophrenia patients and 74 healthy controls from the COBRE dataset, targeting at the local graph theoretical properties of the embedded FCN. Thus, we matched the different anatomical regions of each individual in accordance with the Desikan-Killiany Atlas [31]. This atlas has been recently used in a network-based analysis for exploring the dynamic functional core of human brain at a resting state [32]. We constructed FCN by (a) the standard approach, i.e. by-cross-correlating the rsfMRI time series [15], [18], [19] and, (b) using ISOMAP [16], [27], [33] to produce low dimensional embeddings of the correlation matrices. Based on the constructed FCN, we calculated five local graph theoretic measures, namely the participation coefficient, the strength of node, the betweenness centrality, the nodal efficiency and the local clustering coefficient. These measures have been well tried and tested in recent classification studies associated with schizophrenia [15], [18], [19], [34]. LASSO and Random Forest (RF) algorithms were used for feature selection. In order to select the most informative features and to evaluate the final model's performance we used a linear SVM with a double cross validation scheme.

## 2 Materials and methods

### 2.1 Data description

The analysis is based on the Schizophrenia Centers of Biomedical Research Excellence (COBRE) dataset (publicly available at: http://fcon_1000.projects.nitrc.org/indi/retro/cobre.html). The COBRE dataset comprises of high resolution T1 images as well as rsfMRI data from 146 subjects. For the subject 0040075 there were missing rsfMRI data and subsequently it was excluded from further analysis, leaving a total of 71 Schizophrenic patients (Male/Female: 57/14; handedness R/L/B: 59/10/2; age: 38.1 ± 13.9) and 74 healthy controls (Male/Female: 51/23; handedness R/L/B: 71/1/2; age: 35.8 ± 11.5). All subjects were screened prior to any acquisition and exclusion criteria included: history of neurological disorder other than schizophrenia, mental retardation, severe head traumas with more than five minutes loss of consciousness, substance abuse or dependence within the last 12 months. Acquisition protocols were the same for both groups and included a T1-multi-echo-MPRAGE of high resolution (TR/TE/TI = 2530/[1.64, 3.5, 5.36, 7.22, 9.08]/900 ms, flip angle = 7°, matrix = 256×256×176, voxel-size = 1×1×1 mm^3^) and rs-fMRI with the single-shot echo planar imaging technique (TR: 2 s, TE: 29 ms, matrix size: 64×64, 32 slices, voxel-size: 3×3×4 mm^3^).

### 2.2 Preprocessing and signal extraction

The fMRI data preprocessing was carried out using FEAT (FMRI Expert Analysis Tool) Version 6.00, part of FSL (FMRIB's Software Library). The pipeline included motion correction using Fsl's linear registration tools (MCFLIRT) [35], slice-timing correction using Fourier-space time-series phase-shifting, non-brain tissue removal using Fsl's brain extraction tool (BET) [36], spatial smoothing using a Gaussian kernel of 5mm Full Width at Half Maximum (FWHM), grand-mean intensity normalization of the entire 4D dataset by a single multiplicative factor. In order to further refine our data from noise due to motion artifacts, we also employed denoising via ICA AROMA methodology [37] which detects and regresses out noise-related independent components. High-pass filtering at 100Hz was applied after ICA AROMA procedure as it is recommended, in order to better identify motion-related components while at the same time avoiding ringing artifacts [37]. Additionally, a high-pass filter was favoured against a band-pass, as temporal band-pass filtering discards meaningful signal existing in higher frequencies [38].

Finally, FreeSurfer software package version 6.0.1 [39] was utilized to parcellate each individual T1-MPRAGE image into anatomical regions according to Desikan-Killiany (DK) Atlas [31]. Briefly, the FreeSurfer process pipeline included brain extraction, intensity normalization for the reconstruction of gray and white matter boundaries and pial surface extraction to approximately 150.000 vertices per hemisphere. For pairing each hemispheric surface with a spherical template of DK, atlas non-rigid transformations were performed and corrected iteratively until individual cortical folding patterns were matched with cortical geometry across subjects. Finally, parcellation of each individual subject was registered to the rsfMRI space and time series were extracted from 84 cortical and sub-cortical regions in a subject-specific manner. These time series were used for any further analysis.

### 2.3 Construction of FCN using Cross-Correlation

For each pair of time series, here from *M* = 84 anatomical regions of the brain, say **A**_*i*_ and **A**_*j*_, the cross-correlation function (CCF) (see also in [16]) reads:

$$CCF(A_{i},A_{j},l)=\frac{E[(A_{i,t+l}-{\bar{A}}_{i})(A_{j,t}-{\bar{A}}_{j})]}{\sqrt{E[(A_{i,t}-{\bar{A}}_{i}{)}^{2}]E[(A_{j,t}-{\bar{A}}_{j}}{)}^{2}]},$$ (2.1)

where *l* represent the time lag (i.e. shifting in time, both backwards and forwards), and ${\bar{A}}_{i}$ denotes the average value of the entire time series. Here, we considered a maximum of three time lags (the same number of lags considered also in [16]). Hence, for each pair of time series we calculated 7 correlation scores, three of them shifted back in time (e.g. one, two and three time lags), three of them shifted forward in time and one of them (zero lag) being the simple standard linear correlation. The intuition behind this metric is to detect the correlation between brain regions that might have a high correlation value but with a small phase shift. Cross-correlation has long been applied as an fMRI signal processing strategy [40].

For the construction of the FCN connectivity/distance matrices, we used a pseudo-distance metric *d_c_* defined as (see also in [16]):

$$d_{c}(A_{i},A_{j})=1-\underset{l=0,1,2,3}{max}(|CCF(A_{i},A_{j},l)|).$$ (2.2)

However, the connectivity/distance matrices are hardly comparable across subjects, as they are fully connected [16]. Therefore, as a common practice, we set up a thresholding procedure to the (dis)similarity matrices in order to keep the strongest connections of the derived FCN. In order to remove the effect of the variable network density on the calculation of the graph measures across groups, we followed the approach of proportional thresholding (PT) [41]. We examined different levels of PT ranging from 20% to 50% (similar ranges have been also used in recent studies [4], [15], [18]) with a step of 5%. Other approaches of thresholding that have been proposed include data-driven topological filtering based on Orthogonal Minimum Spanning Trees (OMST) with applications in different neuroimaging modalities [42], [43].

### 2.4 Construction of FCN using ISOMAP

ISOMAP is a non-linear dimensionality reduction/manifold learning algorithm that considering a set of *M* objects/observables $x_{1},x_{2},\ldots ,x_{M}\in R^{N}$ produces a low-dimensional data representation $y_{1},y_{2},\ldots ,y_{M}\in R^{p}$, p≪N minimizing the objective function:

$$\sum_{i,j,\;i\neq j}{\left(\;d_{G}(x_{i},x_{j})-d(x_{i},x_{j})\right)}^{2},$$ (2.3)

where $d_{G}(x_{i},x_{j})$ is the shortest path (e.g. the geodesic distance) and $d(x_{i},x_{j})$ is the (dis) similarity obtained (here, the cross-correlation-based pseudo-distance, described in 2.3) between all pairs of points $x_{1},x_{2},\ldots ,x_{M}\in R^{N}$.

Here, the observables **x**_*i*_ are the amplitudes of time series matched to anatomical regions of the brain $A_{i,\;i=1,..M}\in R^{N}$.

The steps of the algorithm that solves the above minimization problem can be described briefly as follows [33]:

- Create a graph G=(V,E), where the vertices *V* are the time series matched to anatomical regions of the brain **A**_*i*_; its connections *E* are created by applying either the *k*-nearest neighbors algorithm or alternatively, a fixed distance among vertices, the so-called *ε*-distance. For example, a connection/link between time series of different brain regions is established if $d_{i,j}\equiv d(A_{i},A_{j})<\varepsilon \;,\;\forall \;i\neq j$. If a link exists between **A**_*i*_, **A**_*j*_, set the weight *w_i_*_,*j*_ of the edge as $w_{i,j}=\frac{1}{d(A_{i},A_{j})}$. Otherwise, if no link exists between **A**_*i*_ and **A**_*j*_, set *w_i_*_,*j*_ = 0. Here, we used the *k* nearest neighbours algorithm [33] exploring a wide range of values of *k* ∈ [6, 20]. Below *k* = 6 some graphs became fragmented while above *k* = 20 each node would have more than 25% of the total nodes as neighbours. This would end up with a very dense graph that geodesic distance is less meaningful (approximately every node would have a very similar and shortest path to any other). Thus, we decided not to explore values of *k* greater than twenty.
- Calculate the shortest paths (geodesic distances) $d_{G}(A_{i},A_{j})$ between all pairs of nodes based on the distances *d_i_*_,*j*_; the calculation of the shortest paths can be done for example, by applying the Dijkstra algorithm [44]. This procedure results to a matrix **D_G_** containing the geodesic distance between all pairs of nodes:
  $$D_{G_{ij}}\equiv d_{G}(A_{i},A_{j})=min\{d_{i,j},d_{i,k}+d_{k,j}\},\;k=1,2,\ldots ,M\;k\neq i,j.$$ (2.4)
- Estimate the new coordinates of the low-dimensional embedded manifold $y_{1},y_{2},\ldots ,y_{M}$ by making use of the multi-dimensional scaling (MDS) algorithm [45] on the geodesic matrix **D_G_** which was calculated in the previous step.

For the new embedded coordinates $y_{1},y_{2},...,y_{M}$ in the low dimensional space, we set up a thresholding procedure (see subsection 2.3) to construct graphs for further analysis.

The selection of the embedding dimension *p* was based on the spectrum of the eigenvalues resulting from the last step of the algorithm (the MDS decomposition on the geodesic distance matrix **D_G_**) (see also in [27]). A gap between some of the first larger eigenvalues and the rest of the spectrum suggests that these few larger eigenmodes extract most of the information related to the distance differences among data points. Thus, it is known that these few eigendimensions are capable of representing information in the low-dimensional space where non-trivial intrinsic properties are revealed [46]. In particular, to justify possible embedding dimensions, we followed the steps below: The eigenvalues were sorted in decreasing order so that $\lambda_{1}\geq \lambda_{2}\geq \lambda_{3}$ ... $\geq \lambda_{M}$. For every subject, the pairwise differences $\lambda_{1}-\lambda_{2}$, $\lambda_{2}-\lambda_{3}$, ... , $\lambda_{M-1}-\lambda_{M}$ were computed. A gap among the average pairwise differences indicates from which point and further, the consideration of another eigendimension has a small contribution to the construction of the embedded FCN. In other words, a gap in the eigenpectrum of the final decomposition denotes the intrinsic dimensionality of the manifold from which the data points were sampled [46]. Another alternative metric that we computed for the selection of the embedding dimension is that of the residual variance $1-R{\left(D_{G},D_{Y}\right)}^{2}$ [33], where *D_Y_* is the matrix of euclidean distances in the low dimensional embedding, *D_G_* the matrix with the geodesic distances (the algorithm's best estimate of the intrinsic distances) and *R* the Pearson correlation coefficient. Again, a gap after the first few embedding dimensions in the residual variance is indicative of the intrinsic dimension of the dataset [33]. For the implementation of the ISOMAP algorithm, we utilized the package “vegan” [47] in the R free software environment [48].

### 2.5 Local graph-theoretical measures

We assessed five key local graph measures as described in [49]. In particular, we analyzed the local topological properties of the obtained FCN as resulted through thresholding on the basis of the strength of node, the betweenness centrality, the local efficiency, the local clustering coefficient and the participation coefficient [49]. The final feature vector size for each subject was 420-dimensional (5 local properties for each one of the 84 nodes of an individual's graph). Given a graph G=(V,E) where *N* is the total number of nodes, *a_ij_* represents the binary link (0, unconnected or 1, connected), $k_{i}=\sum_{j\in N}a_{ij}$ is the degree of node and *d_ij_* the distance that separates node *i* from node *j*, the above graph measures are defined as follows [49]:

- Strength of node: $s_{i}=\sum_{j\in N}a_{ij}w_{ij}$, where *w_ij_* is the weight of an edge coincide with node i and j (here, links between nodes represent distances, so $w_{ij}=\frac{1}{d_{ij}}$).
- Local efficiency: $E_{l_{i}}=\frac{1}{N_{G_{i}}}\sum_{i\in G}E_{g}(G_{i})$, where $N_{G_{i}}$ is the number of the nodes of the subgraph **G**_*i*_ that is consisted of the node's neighbors. *E_g_* is the global efficiency of a graph given by $E_{g}=\frac{1}{N(N-1)}\sum_{i\neq j\in G}\frac{1}{D_{G_{i,j}}}$, where M is the number of nodes and $d_{G_{i,j}}$ are the shortest paths between nodes i and j.
- Local clustering coefficient: $c_{i}=\frac{1}{s_{i}(k_{i}-1)}\sum_{jh}\frac{w_{ij}+w_{ih}}{2}a_{ij}a_{ih}a_{jh}$.
- Betweenness centrality: $b_{i}=\sum_{i\neq j\neq m}\frac{n_{mj}(i)}{n_{mj}}$, where $n_{mj}(i)$ is the number of shortest paths between nodes *n_mj_* that pass through node i and *n_mj_* is the total number of shortest paths.
- Participation coefficient: $PC_{i}=1-\sum_{m\in M}{\left(\frac{k_{i}(m)}{k_{i}}\right)}^{2}$ where **M** is the set of modules, *k_i_*(*m*) is the degree of node *i* inside the module m. The so-called “Louvain algorithm” [50] was employed for finding the community structure of the graph.

The strength of a node is a fundamental measure of prominent importance that can characterize the centrality of a node. Nodes with high strength are often considered as main hubs of the network [49]. An alternative to the strength is the betweenness centrality score, that can also reflect the degree of centrality from another perspective. A high betweenness centrality score for a node indicates that most of the information that flows within a network passes through that node. The local clustering coefficient is another key measure of functional segregation of a node [51]. It quantifies the interconnection of node's neighbours or the possibility that nearby neighbours of a node have also links to each other. The local efficiency reflects the ability of a sub-network constructed by a node and its immediate neighbours to transfer information in terms of the shortest path lengths. For example, a high local efficiency score indicates that the information transmission is more efficient/faster since the shortest paths between them are small. The participation coefficient measures a node's distribution of links/edges among different modules/communities of the graph. When a node doesn't share any link outside its module, then the participation coefficient is zero. On the other hand, if the edges of a node distribute evenly between all modules of a graph, then the coefficient reaches its maximal value. Finally, one can characterize a node based on its participation coefficient as a “provincial” hub, if the score is low, or as a “connector” hub if the score is high [52].

The graph measures were computed utilizing the “igraph” [53] and “brainGraph” packages in the R free software environment [48].

### 2.6 Feature selection

Here, we used two different methodologies/strategies, namely the Least Absolute Shrinkage and Selection Operator (LASSO) and Random Forests (RF) for feature selection. Our final feature matrices were of high dimensionality (the final feature vector size was 420-dimensional: 5 measures for each of the 84 nodes matched to the different anatomical regions of the brain) containing a lot of redundant information. Thus, the choice of the most predominant features is crucial for the classification process and the detection of potential biomarkers of the disease.

#### 2.6.1 Least Absolute Shrinkage and Selection Operator (LASSO)

The LASSO algorithm has been utilized in recent studies on schizophrenia for both accurate classification and detection of biomarkers of the disease [15], [54].

The procedure of the LASSO algorithm stems by finding a solution to the following optimization problem:

$$\underset{a}{argmin}∥y-Xa∥+\lambda_{1}{∥a∥}_{1},$$ (2.5)

where **X** represents the feature matrix of size *n*×*p* with *n* being the number of subjects and *p* the dimensionality of the feature vector, **y** is a vector containing the class labels for the subjects (0 for healthy controls, 1 for schizophrenia patients), **a** is the vector of coefficients and *λ*_1_ the penalizing factor. The penalty factor *λ*_1_ determines how regularized the model is, and thus, how many features are retained. As the value of the penalizing factor drops, coefficients are getting smaller and smaller. Typically, most of them decline to zero. The absolute value of a non zero coefficient can be then used as a measure of the feature's relative importance. It is highly recommended to use LASSO in conjunction with cross-validation [26], [55], [56] (here we used the LOOCV scheme). Thus, one can choose the penalizing factor based on the misclassification error estimated by a CV procedure, so that it is more probable that the final model generalizes well to unknown data samples. Here, we estimated the CV classification error and chose the penalizing factor according to the “one standard deviation rule”. According to this rule [24], we selected the most parsimonious model with its error being no more than one standard deviation above the error of the best model. This practice has been suggested by Hastie *et al*. [57] for general cross-validation use. For the implementation of LASSO, in conjunction with cross validation, we utilized the “glmnet” package [56] of the R free software environment [48].

#### 2.6.2 Random forest (RF)

RF is an ensemble machine learning algorithm used for both classification and regression purposes [58]. It comprises of a large number of classification and regression trees [59], where each one operates independently. Every individual tree is constructed using a bootstrap (i.e. random sampling with replacement) version of the training data. Once the tree is constructed, the “out of bag” sample (the instances not used in the construction step) of the original data is used as a test set. The error rate in the “out of bag” samples of all trees in the forest is the estimate of the generalization error of the final model. Prediction of new samples are made through a majority voting system between trees where the class with the most votes becomes the final prediction of the model. While the algorithm is inherently stochastic is considered to be robust to noise and resistant to both overfitting and the presence of outliers [60]. The algorithm has been applied to fMRI data for both feature selection and classification purposes [61], [62]. RF, can be also utilized for feature selection using random subspace methodology for measuring feature importance by calculating the so-called Gini impurity index [63]. This index/criterion is computed based on the impurity reduction principle [64] and make no hypothesis of data belonging to specific distributions and therefore,is non parametric. For a binary split, the Gini index of a node *t* can be calculated as follows:

$$G(t)=1-\sum_{j=1}^{2}p{\left(j\right)}^{2},$$ (2.6)

where *p* is the frequency of each class *j* that passes through that node. A low Gini index implies that the specific feature is important in partitioning data into the two distinct classes (here, schizophrenia patient/healthy control). Specifically, a tree structure 𝒯 trained on a learning sample of size *N* tries to identify at each node *t*, a split *s_t_* for which the sample *N_t_* that pass through the node is split into two child nodes *t_R_* and *t_L_* by maximizing the decrease below [65]:

$$\Delta G(s,t)=G(t)-p_{R}G(t_{L})-p_{L}G(t_{R}),$$ (2.7)

where $p_{L}=N_{t_{L}}/N_{t}$,$p_{R}=N_{t_{R}}/N_{t}$. Adding all the weighted decreases of all nodes *t* using a specific feature, say *X_m_* averaged over all trees, one can obtain the mean decrease in Gini index (MDG) [65]:

$$MDG(X_{m})=\frac{1}{N_{T}}\sum_{T}\sum_{t\in T:u(s_{t})=X_{m}}p(t)\Delta G(s_{t},t),$$ (2.8)

where *N_T_* is the number of trees in the forest, $p(t)=N_{t}/N$ and *u*(*s_t_*) is the feature used in the split of node *t*.

MDG reflects the average of a variable's total decrease in node impurity, weighted by the proportion of samples reaching that node across all trees of the ensemble. In order to have a robust evaluation of feature importance it is generally recommended that the RF should be run multiple times [66]. After measuring and ranking features by importance, elimination of bottom less useful features may lead to an increase in the accuracy of the final model [61]. In this study, we perform feature selection via RF in two steps:

- We estimated MDG for all features across 30 independent runs (more runs did not change the outcome of the analysis) of the model. We averaged the results to eliminate the stochastic nature of the algorithm in order to obtain a stable ranking. We eliminated 95% of the features and kept only the most important ones (the ones with the highest MDG indices).
- For the remaining ranked features, we trained models with different subsets of predictors starting from the single most important feature to all features remained after elimination, by a step of two (the first model uses only one feature, the second the first three, the third the first five etc.). In the case that two or more models were tied in terms of performance, we chosen the simplest model (the one with the fewer features).

For our computations, we used the R package “randomForest” [67]. The number of trees in the “forest” was set to 500 (similar number of trees also used in [61], [62]) and the parameter concerning the number of features analyzed at each node to find the best split was set equal to the square root of the number of features. The latter is the recommended value of the package and the same strategy have been also used by other studies [61], [62], [66].

### 2.7 Overview of the methodology and classification procedure

**Figure 1.**
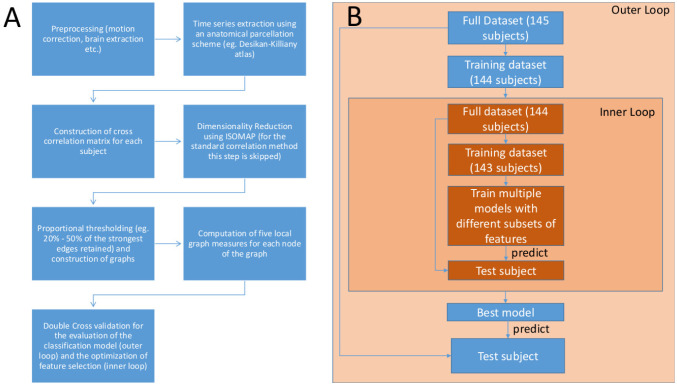
A) Overview of the proposed methodology. B) The double cross validation scheme.

The overall pipeline proposed in this study (1A) along with the classification scheme (1B) used is presented in Figure 1. At first, we pre-processed the raw fMRI data (see 2.2) and extracted the time series using an anatomical parcellation scheme (the Desikan-Killiany atlas) for each subject (see 2.2). As a next step, we constructed the cross correlation matrices of the derived time series (see 2.3). We then embedded the dimension of the matrices using ISOMAP and constructed the graph objects of the low dimensional embeddings (this step is skipped in the case of the correlation method). We then applied proportional thresholding (PT) on the graph structures and computed the 5 key local graph-theoretic measures (see 2.5) as described in the methodology. To ensure that the selected features can be generalized across subjects [20], and that the evaluation of the model's performance will be unbiased [24], we performed a double cross validation procedure [19]. Our double cross validation consisted of an outer and an inner loop of “leave one out” cross validation (LOOCV) scheme. The outer loop evaluates the model's performance, while the inner loop optimizes the feature selection procedure (here with LASSO and RF). Initially, out of the total number of subjects considered in this study (*N* = 145), we first left one subject out (as test subject) and continued with the other 144 (for training and validation). On these remaining subjects (*N* = 144) we employed the feature selection with another (inner) loop of LOOCV. We then trained a linear standard SVM with the features determined by the inner loop of LOOCV and tried to predict the class label of the initially left/unseen test subjects. This procedure is repeated 145 times. Thus, the estimation of the final model's performance remains unbiased and the selected features are more likely to be generalizable for unseen samples [19], [20]. A schematic representation of the procedure discussed above can be inspected in 1B.

The confusion matrix was also computed for the classification model. In the case of binary classification, the confusion matrix is a 2 × 2 square matrix reporting the number of true positives *TP*, false positives *FP*, true negatives *TN* and false negatives *FN*. In particular, we considered cases of schizophrenia patients as positives *P* and healthy controls as negatives *N*. Sensitivity (known also as the True Positive Rate) and specificity (True Negative Rate) are statistical measures for the evaluation of a binary classification model. The sensitivity *TPR* is defined as $TPR=\frac{TP}{TP+FN}$, while specificity *TNR* as $TNR=\frac{TN}{TN+FP}$. Here, Specificity quantifies the ability of a model to correctly identify a healthy control subject, while sensitivity measures the proportion of schizophrenic subjects correctly identified by the model.

### 2.8 Support vector machines (SVM)

The classification performance was assessed in the outer loop of LOOCV using standard Linear Support Vector Machines (LSVM). The features given to the classifier were those features determined by the optimization of feature selection procedure inside the inner loop of LOOCV. The final “best” LSVM model was trained 145 times on 144 out of 145 subjects and each time we tried to predict the class label of the unseen test subject. Briefly, the LSVM finds the best plane or hyperplane that separates the two groups in the feature space. Specifically, given a set of data points ${\left(x_{i},y_{i}\right)}_{i=1,2..N}$, where *N* reflect the total number of subjects, $x_{i}\in R^{d}$ represents *d* attributes/features for subject *i* and $y_{i}\in (-1,1)$ the subject's class label (here, a schizophrenia patient or a healthy control), SVM aims at finding the best separating hyperplane by maximizing the margin of separation. In general, a hyperplane can be defined as $w\cdot x_{i}+b=0$ where **w** are the weights of features **x**_*i*_. Any hyperplanes lying in parallel can then be described as $w\cdot x_{i}+b\geq 1$ if *y_i_* = 1 and $w\cdot x_{i}+b\leq -1$ if *y_i_* = –1. Thus, the optimization problem refers to the maximization of the margin between hyperplanes $\frac{2}{∥w∥}$ so that for every ${\left(y_{i}\right)}_{i=1,2..N},\;y_{i}\cdot (w\cdot x_{i}+b)\geq 1$. A regularization parameter *C* is also included. This parameter controls the penalty of the error *z_i_* and allows for a trade-off between misclassifications and size of the separating margin. For example, a large value of *C*, will end up with a smaller-margin between separating hyperplanes. On the other hand, a small value will lead the solution of the final optimization problem to a larger-margin, even if that hyperplane misclassifies some points (in order for the model to generalize well in future data samples). Therefore, the final optimization problem, becomes the one which minimizes $\frac{∥w{∥}^{2}}{2}+C\cdot \sum_{i}z_{i}$ subject to $y_{i}\cdot (w\cdot x_{i}+b)\geq 1-z_{i}$, i=1,2..N. Here, The regularization parameter *C* was set to 1 (similar value of parameter *C* used also in [19]). For the implementation of SVM algorithms, we utilized those offered by the “caret” [68] package of the R free software environment [48].

## 3 Results

Following the methodology described in section 2.3, we first constructed the FCN by correlating the time-courses (matched to the 84 anatomical brain regions) and then we proceeded with the construction of FCN based on the ISOMAP.

### 3.1 Construction of low dimensional embeddings of FCN using ISOMAP and visualization

**Figure 2.**
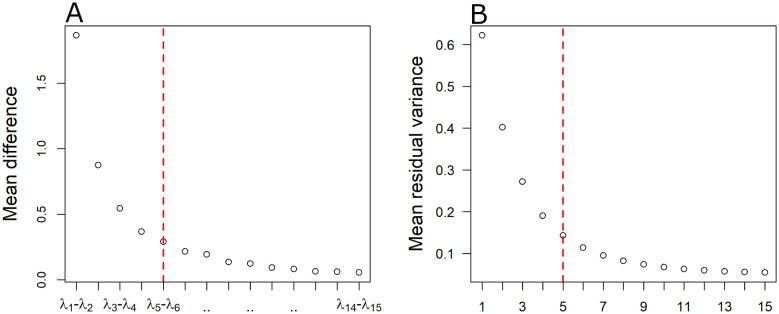
A) Average pairwise differences of the 15 largest eigenvalues (see 2.4). B) Mean residual variance for low dimensional embeddings of different dimensionality (1 to 15 dimensions) as derived by ISOMAP based on the optimal parameters. The red dashed vertical line marks the maximum number of dimensions.

**Figure 3.**
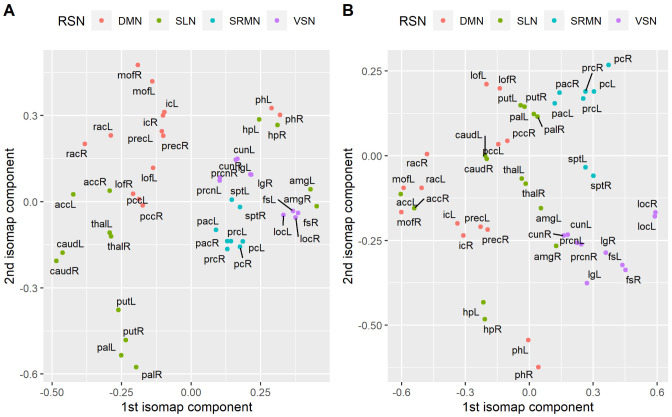
Visualization of a 2D embedding given by ISOMAP on the average correlation matrix for each group. Here, only brain regions that are considered part of four major RSNs, the default mode (DMN), the sensory motor (SMRN), the visual (VSN) and the sub-cortical limbic network (SLN) are included. A) Healthy controls B) Schizophrenia patients. The capital letter R and L at the end of each label reflects the lateral part of the brain region (eg. L for left and R for right). The list of the abbreviations used to label the anatomical regions of the brain is the following. For the DMN: isthmus of cingulate gyrus (ic), lateral orbito-frontal cortex (lof), medial orbito-frontal cortex (mof), parahippocampal gyrus (ph), posterior cingulate cortex (pcc), Precuneus cortex (prec), rostral anterior cingulate cortex (rac). For the SLN: accubens (acc), amygdala (amg), caudate nucleus (caud), pallidum (pal), putamen (put) and thalamus (thal). For the SRMN: paracentral gyrus (pac), postcentral gyrus (pc), precentral gyrus (prc) and superior parietal (sp). For the VSN: Cuneus (cun), fusiform gyrus (fs), lateral Occipital cortex (loc), lingual gyrus (lg) and pericalcarine cortex (prcn).

As described in the methodology, the embedding dimension was selected via the inspection of the eigenspectrum of the decomposition and complementary by inspecting the residual variance for each one of the low dimensional embeddings. In Figure 2A, we show the average differences between the fifteen largest eigenvalues of the final decomposition, while in 2B the average residual variance across subjects. As it is shown in Figure 2A, there is a large gap between the first and second pair of eigenvalues, while a second gap appears between the second and the third eigenvalues. Finally, a smaller third gap appears between the third and fourth pair of eigenvalues. Finally, after the fifth and sixth eigenvalues, most of the pairwise differences have an almost equal value that is close to zero. Similarly, the average residual variance (Figure 2B) falls rapidly until the first 3 dimensions and continues decreasing smoother and smoother up to five dimensions. In fact, this indicates that there is no substantial difference in the magnitude of eigenvalues (and the decrease in the residual variance) further from this point. Thus, we decided not to explore low-dimensional embeddings larger than five dimensions. Instead, we focused our analysis on four low-dimensional embeddings where 2,3,4 and 5 dimensions are retained. In Table 1, we report the residual variance per embedding dimension for each group. In Figure 3, we present a 2D low dimensional embedding of the average correlation matrix for each one of the two groups (healthy controls and schizophrenia patients) as derived by ISOMAP. For visualization purposes, we included only 46 out of the 84 brain regions which belong to four major Resting state networks namely the Default Mode Network (DMN), the Sub-cortical Limbic Network (SLN), the Sensory Motor Network (SRMN) and the Visual Network (VSN). We label as DMN the lateral parts of the isthmus of cingulate gyrus (ic), lateral orbito-frontal cortex (lof), medial orbito-frontal cortex (mof), parahippocampal gyrus (ph), posterior cingulate cortex (pcc), Precuneus cortex (prec), rostral anterior cingulate cortex (rac). SLN includes the lateral parts of the accubens (acc), amygdala (amg), caudate nucleus (caud), pallidum (pal), putamen (put) and thalamus (thal). SMRN comprises of lateral parts of paracentral gyrus (pac), postcentral gyrus (pc), precentral gyrus (prc) and superior parietal (sp). Finally VSN includes lateral parts of the Cuneus (cun), fusiform gyrus (fs), lateral Occipital cortex (loc), lingual gyrus (lg) and pericalcarine cortex (prcn). As it can be seen from Figure 3, ISOMAP achieves a satisfactory grouping of the four major RSNs in the case of controls 3A. On the other hand nodes are more disorganized in the case of patients with schizophrenia 3B.

**Table 1.** Residual variance per Embedding dimension for each group (eg. healthy controls and schizophrenia patients).

Group	Emb. dimension	Residual variance (Mean ± SD)
Healthy controls	*p* = 1	0.61 ± 0.11
	*p* = 2	0.39 ± 0.12
	*p* = 3	0.25 ± 0.1
	*p* = 4	0.18 ± 0.08
	*p* = 5	0.13 ± 0.06

Schizophrenia patients	*p* = 1	0.63 ± 0.12
	*p* = 2	0.41 ± 0.12
	*p* = 3	0.29 ± 0.11
	*p* = 4	0.21 ± 0.08
	*p* = 5	0.15 ± 0.07

### 3.2 Classification performance

Using LASSO, the best, with respect to the embedding dimension, number of *k* nearest neighbors in the ISOMAP algorithm and the level of proportional thresholding (PT), classification rates along with the sensitivity and specificity rates for each method are shown in Table 2. Similarly, results using the RF method for feature selection are also shown in Table 3.

**Table 2.** Best classification rates obtained with the two methods employed in this study; proportional thresholding PT, embedding dimension *p*, parameter (*k*-nearest neighbours for ISOMAP), accuracy (Acc), sensitivity (Sens), specificity (Spec) rates. Features were chosen by LASSO.

Method	Emb.dim	Parameter	PT (%)	Accuracy (%)	Sensitivity (%)	Specificity (%)
Correlation	-	-	30	**73.1**	77.4	68.9

ISOMAP	*p* = 2	*k* = 14	40	75.9	83.1	68.9
	*p* = 3	*k* = 15	35	**79.3**	85.9	72.9
	*p* = 4	*k* = 12	40	76.6	85.9	67.6
	*p* = 5	*k* = 18	45	75.9	80.3	71.6

**Table 3.** Best classification rates obtained with the two methods employed in this study; proportional thresholding PT, embedding dimension *p*, parameter (*k*-nearest neighbours for ISOMAP), accuracy (Acc), sensitivity (Sens), specificity (Spec) rates. Features were chosen by RF.

Method	Emb.dim	Parameter	PT (%)	Accuracy (%)	Sensitivity (%)	Specificity (%)
Correlation	-	-	40	**71**	77.4	64.8

ISOMAP	*p* = 2	*k* = 15	45	76.6	80.3	72.9
	*p* = 3	*k* = 15	35	**78.6**	87.3	70.3
	*p* = 4	*k* = 12	40	74.5	81.7	67.6
	*p* = 5	*k* = 17	45	76.6	81.7	71.6

**Figure 4.**
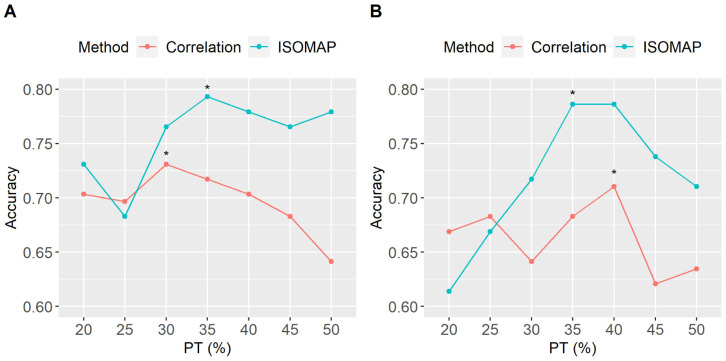
Classification performance across all thresholding (PT) points for the two methods employed (cross-correlation and ISOMAP). A) When LASSO used for feature selection. B) when RF algorithm was used for feature selection. The PT points with the best classification rates are marked with an asterisk “*”. Using LASSO for feature selection, ISOMAP peaked at 79.3% with a 35% PT while cross-correlation peaked at 73.1% with 30% PT. Using the RF methodology would decrease the highest classification rates for both ISOMAP and correlation by almost 1% and 2% respectively.

**Figure 5.**
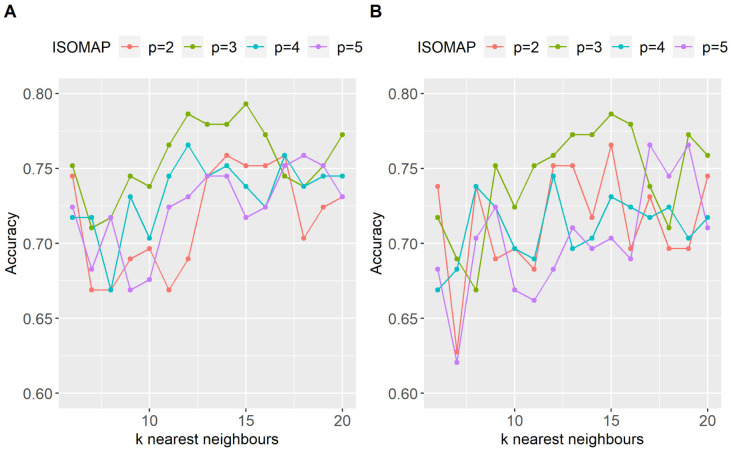
Classification performance for different values of the *k*-nearest neighbours of ISOMAP when A) features selected by LASSO, B) features selected by RF. Accuracy rates are shown for all 4 different low-dimensional embeddings (*p* = 2,3,4,5)

Using LASSO for feature selection, the best classification rate for ISOMAP was 79.3%, obtained retaining 3 dimensions, *k* = 15 nearest neighbours and 35% PT. The conventional correlation peaked at 73.1% (at 30% PT). Interestingly, the 6.2% difference in the classification performance translates mainly to an increase in the sensitivity (reflecting the ability of the model to correctly identify a schizophrenic subject). Using RF for feature selection the results were similar to those obtained with LASSO. Specifically, the correlation method peaked at 73% (at 40% PT) while ISOMAP at 78.6% and 35% PT. The whole pattern of classification rates, using both strategies of feature selection, through all PT points used in this study is shown in Figure 4A,B. Using both feature selection methodologies, ISOMAP did not only produced the best classification rates but was also more robust with respect to the level of thresholding when compared to the standard methodology. When using LASSO 4A, ISOMAP was more robust than the correlation method for a wide range of levels of PT (eg. 30%–50%) with the accuracy rates being above 75%. When using RF for feature selection, ISOMAP provided again superior results with accuracy rates being higher than 70% in the PT range of 30% to 50%. Figure 5 depicts the maximum classification obtained for each low dimensional embedding (2, 3, 4 and 5 dimensions retained) with respect to the value of *k* nearest neighbors for both of the feature selection strategies employed in this study (eg LASSO and RF). All four ISOMAP-based low-dimensional embeddings produced better classification rates than the standard cross-correlation, while the overall optimal was obtained when using three embedding dimensions for both feature selection methods.

### 3.3 Features selected using LASSO

**Figure 6.**
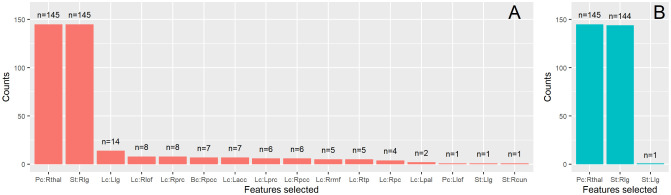
Features selected when utilizing LASSO methodology for A) The correlation method, B) ISOMAP. The total number of computational experiments were 145 in total. Thus, at maximum a feature can be selected n = 145 times. The x-axis shows the features that each method selected at least once. The letters Pc, Lc, Bc and St (before the colon “:”) denote the participation coefficient, local clustering, betweenness centrality and strength, respectively (neither of the methods chose any node's local efficiency). The capital letter after the colon refer to the lateral part of the anatomical region (Left and Right lateral parts) of the node and finally the remaining letters denote the region itself. The list of the abbreviations concerning the anatomical regions of the brain is the following: acc: accubens nucleus; cun: cuneus; lg: lingual gyrus; lof: lateral orbito-frontal cortex; pal: pallidum; pc: post central gyrus; pcc: posterior cingulate cortex; prc: pericalcarine; rmf: rostral middle frontal gyrus; tp: temporal pole; thal: thalamus.

Despite the fact that the final feature vector for each subject was 420-dimensional (five local measures for each one of the 84 brain regions) with the total number of subjects being 145, the LASSO selected mainly, two features. The selected features which were chosen almost equally for both ISOMAP and correlation were the participation coefficient of the right thalamus and the strength of the right lingual gyrus. In Figure 6, are shown barplots of all the features chosen (at least once) for each method considering the optimal parameters (proportional thresolding (PT) point, embedding dimension and *k*-nearest neighbours) that produced the highest classification rates. Thus, the maximum number of selections for a feature would be 145 (total number of the independent experiments LOOCV with a sample of 145 subjects). When using correlation (6A), LASSO selected mainly two features (the participation of the right thalamus and the strength of the right lingual gyrus), but in some cases would end up with a more complex model, adding more features. The absolute coefficients of the LASSO model over 145 computational experiments were $a_{Pc:Rthal}=0.08\pm 0.05$ for the participation coefficient of right thalamus and $a_{St:Rlg}=0.18\pm 0.05$ for the strength of right lingual gyrus. When using ISOMAP (6A), LASSO selected almost exactly these 2 features (the participation coefficient of the right thalamus with average coefficient $a_{Pc:Rthal}=0.28\pm 0.04$ and the strength of the right lingual gyrus, $a_{St:Rlg}=0.11\pm 0.04$) in every computational experiment. Therefore, using ISOMAP the participation coefficient of the right thalamus considered more important for LASSO than the strength of the right lingual gyrus. The exact opposite was observed when using the correlation method.

### 3.4 Features selected using Random Forests

**Figure 7.**
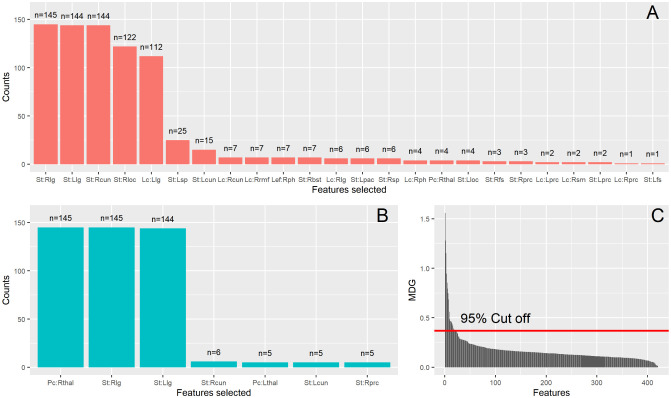
Features selected when using Random Forests (RF) (see 2.6) using A) the standard correlation method, and B) ISOMAP. The total number of computational experiments were 145 in total. Thus, at maximum a feature can be selected n = 145 times. The x-axis shows the features that each method selected at least once. The letters Pc, Lef, Lc, Bc and St (before the colon “:”) denote the participation coefficient, local efficiency, local clustering, betweenness centrality and strength, respectively (neither of the methods chose any node's betweenness centrality). The capital letter after the colon refers to the lateral part of the anatomical region (Left and Right lateral parts) of the node and finally the remaining letters denote the region itself. The list of the abbreviations of the anatomical regions of the brain is the following: bst: banks of the superior temporal sulcus; cun: cuneus; fs: fusiform gyrus; lg: lingual gyrus; loc: lateral occipital cortex; pac: paracentral gyrus; prc: pericalcarine; ph: parahippocampal gyrus; rmf: rostral middle frontal gyrus; sm: supramarginal gyrus; sp: superior parietal; thal: thalamus. C) Characteristic ranking of feature importance over 30 RF model runs inside the inner loop of LOOCV (see 2.6). The ranking is based on the Mean Decrease Gini (MDG) for each feature. The red horizontal line marks the cut off value used for collecting the most important features.

In general, when using the Random Forest (RF) algorithm for feature selection, more features were selected comparing to LASSO. Barplots of selected features when using the RF algorithm are presented in Figure 7. Specifically, the features selected were 24 when using the cross-correlation method (7A) and 7 when using ISOMAP (7B). Most selected features (selected more than 100 times) for the correlation method were the strength of the right and left lingual gyrus (145 times $MDG_{St:Rlg}=1.6\pm 0.06$ and 144 times $MDG_{St:Llg}=1.28\pm 0.06$ respectively), the right cuneus (144, $MDG_{St:Rcun}=1.14\pm 0.05$), the right lateral occipital cortex (122, $MDG_{St:Rloc}=0.96\pm 0.05$) and local clustering of the left lingual gyrus (112, $MDG_{Lc:Llg}=0.84\pm 0.05$). When using ISOMAP, the most frequently selected features were the participation coefficient of the right thalamus (145 times, $MDG_{Pc:Rthal}=1.91\pm 0.06$) and strength of the right and left lingual gyrus (145 times, $MDG_{St:Rlg}=1.59\pm 0.06$ and 144 times, $MDG_{St:Llg}=1.5\pm 0.06$ respectively). ISOMAP provided more simple models for classification achieving better accuracy. It is remarkable that almost the same features were chosen when using ISOMAP regardless of the method used for feature selection. Thus, when using ISOMAP, the highest classification rates obtained were almost identical for both feature selection strategies (79.3% for LASSO and 78.6% for RF). Interestingly, when using the cross-correlation method 7B, the participation coefficient of right thalamus was chosen only few times (four times). The Random Forest algorithm could not identify the importance of these features; it added other features in the model, ultimately leading to lower classification rates. A characteristic ranking of features over 30 RF model runs can be seen in Figure 7C. The ranking was based on the Mean Decrease Gini (MDG) index 2.6 and shows a great amount of redundancy inherent to the dataset. Thus, only a small set of features were identified as important for the classification between healthy controls and patients with schizophrenia.

### 3.5 Most important features detected

**Figure 8.**
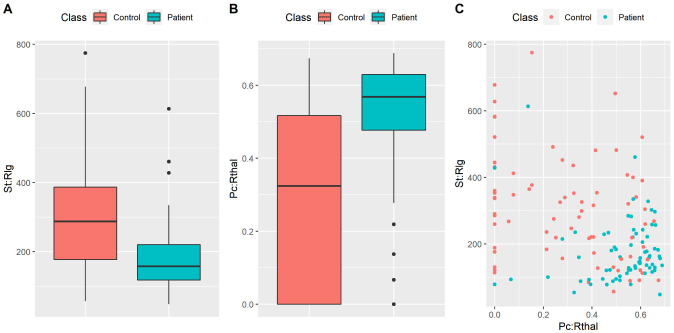
Boxplots of A) the strength of the right lingual gyrus (St:Rlg), B) the participation coefficient of the right thalamus (Pc: Rthal). C) 2D Projection of subjects on the two most important features reported in this study (eg. the ones yielded the best classification accuracy rates).

Finally, for the two most important features (the ones derived by ISOMAP and led to the highest accuracy rate ), we present the distributions between groups as boxplots (Figure 8A,B). Specifically, it is shown that controls exhibited a larger strength in the right lingual gyrus (Welch's t-test: p < 0.05 Bonferroni corrected for multiple comparisons over 420 features) while patients exhibited larger participation coefficients of the right thalamus (Welch's t-test: p < 0.05 Bonferroni corrected for multiple comparisons over 420 features). We also show a projection of each subject on a 2D feature space as derived by these 2 features (Figure 8C). Despite the fact that it is clear that these two features provide a good classification between groups, the cluster of patients is more tight than the cluster of healthy controls. This observation indicates that our model tends to provide a relatively high sensitivity rate and a more modest specificity rate.

## 4 Discussion

Our analysis targeted at revealing classification biomarkers for schizophrenia patients at the ROI level based on resting state fMRI recordings using ISOMAP for the construction of embedding FCN. For our analysis, we used the publicly available dataset (COBRE) matching the brain anatomies to the Desikan-Killiany brain atlas. To assess the classification performance of the FCN constructed with ISOMAP, we compared it against the standard cross-correlation technique on a double cross-validation basis. When using the standard cross-correlation approach, we got similar results with the ones reported in previous studies [18], [19], [69], [70]. For example, Moghimi et al. [19] used correlation to analyse a similarly large independent rsfMRI dataset of 170 subjects (88 healthy controls and 82 schizophrenia patients) using double cross-validation for both feature selection and model validation, getting classification rates as high as 73%. In the study of [19] that employed the correlation method for constructing FCN from rsfMRI matching to anatomical regions of the brain (as in our work), the highest classification rate obtained using a single feature. In particular, this feature was the matching index between the left thalamus and the left post central gyrus; the thalamus is part of the reward system while the post central gyrus is located in the primary somatosensory cortex, which is part of the somatosensory system. The best accuracy rate was 73% and in the same threshold point as the one we found here (preserving 30% of the strongest edges). Moreover, the sensitivity and specificity rates reported in [19] were close enough with the ones reported here when using the cross-correlation method and LASSO for feature selection. In particular, they reported a sensitivity rate of 77% (here, 77.4%) and specificity rate of 68% (here 68.9%) using SVM. This is remarkable, as we worked with an independent dataset and tested different set of features utilizing different feature selection methods. In a recent study [21], the authors applied a classification framework proposed in [22] to two independent datasets evaluating the within and between site generalizability of the model. While the proposed methodology in [22] reported a satisfying 93% accuracy rate, Cai et al. [21] found a within-site accuracy rate of 73% and between-site accuracy of 70%. The approximately 20 % difference was also found in the study of [19] that is attributed to the differences of the results taken when using single and double cross validation. Single cross validation tended to over-optimistically evaluate the model's performance [19], [20]. Using the standard cross-correlation and double cross validation we report practically the same classification accuracy with the one reported in [19] working on an independent dataset. In another study of Nieuwenhuis et al. [69], two large independent datasets were analysed. Using T1 structural MR images and double cross-validation, they reported a 70.4% classification accuracy. Another more recent study [71] involving also two large independent fMRI datasets tested different strategies of Group Independent component analysis for the derivation of subject specific networks from group networks. They used these networks as features and the best reported average classification accuracy was 76.5% (evaluating the model with 100 repeats of cross validation). These classification rates are more likely to reflect the true classification power that contemporary models have [20], [21]. [23]–[25].

Our feature selection analysis based on both the LASSO and Random Forest algorithms revealed that out of a 420-dimensional feature vector for each subject, only a handful of features were important. Thus, there was a great amount of redundancy inherent to the dataset, a fact that was also reported in [19] (where just one single feature produced the best accuracy rates), in [70] (where only seven discriminative independent components used as features) and in [18] (where ten features were found as important, namely the betweenness centrality scores of ten prominent hubs of the network).

On the other hand, ISOMAP has been recently applied for the construction of FCN [16], [27] with the nodes of the network matching the outcomes of a single-subject ICA analysis. [16], working on the same dataset as here, reported a 65% classification accuracy using SVM, considering 13 global graph theoretic measures (no feature selection was performed, thus all these measures were given as features to the classifier). The model's performance was evaluated using a 10-fold cross validation scheme. Here, building on this work and on a recently published work (see [27]) which targeted at the global properties of the FCN using ICA, we made a more thorough assessment of ISOMAP, focusing on the analysis of the local properties of the embedded FCN, thus being able to identify classification biomarkers linked with the activity in specific brain regions. First, we used an anatomical atlas to associate the rsfMRI time series with specific brain regions in order to address the well known problem of the variability in the ICA decomposition when used for the construction of FCN [27], [72]. The experiment comprised of only a single session per subject with a relatively small duration (6 minutes), so we wouldn't expect a robust decomposition for all subjects (see also the discussion in [73]). Second, for our analysis we used key local graph theoretic-measures (strength of node, local clustering, local efficiency, participation coefficient and betweenness centrality) and performed feature selection using LASSO and Random Forests. Third, we considered a wide range of values for tuning the parameter the number *k* of nearest neighbours in ISOMAP and justified the choice of the low-dimensional embedding dimension based on the gap in the eigenspectrum and the residual variance. Fourth, we took an “extra” advanced pre-processing step by using ICA AROMA [37] to detect and factor out noise-related (motion artifacts and other structured noise components like cardiac pulsation confounds) independent components as it is highly recommended (see the discussion in [16]). Fifth, we compared ISOMAP to the current standard cross-correlation (by correlating the time series and performed thresholding to construct FCN), based at the local theoretical graph measures of the derived FCN.

Our analysis showed that ISOMAP outperformed the standard cross-correlation approach, thus increasing the classification performance by 6.2%. This difference was translated mainly to an increase in the sensitivity rate (the ability of a classification model to correctly identify a schizophrenic subject). The sensitivity rate for ISOMAP was 85.9% compared to a 77.4% for the conventional method. Furthermore, the performance of ISOMAP was more robust producing higher classification rates than the conventional methodology across different threshold points. As ISOMAP needs to be tuned (*k*-nearest neighbours, embedding dimension *p*), we showed that for a considerable range of the values of *k*, ISOMAP scored higher classification rates (above 75%). All low-dimensional embeddings produced higher classification rates than the competent standard approach. Finally, when using ISOMAP, both of the feature selection methods had a tendency to choose a more simple model (with mainly two or three features, appearing in almost all 145 computational experiments). This indicates that ISOMAP captured most of the useful information in the dataset (by reducing it to its intrinsic dimension), while discarded information that could be attributed to various sources of noise and confounds (see also the discussion in [46]). At this point, we should note that we decided to use a simple “baseline” classifier (such as the LSVM) as the aim of this study was the investigation of the performance of ISOMAP compared to the standard cross-correlation and not the influence of various machine learning approaches. Consequently, our analysis was focused on how the ISOMAP-based derived embedded FCN affect the feature selection procedure as implemented here by Lasso and Random Forests. Finally, we used a double cross-validation scheme to get an unbiased estimate of the expected accuracy of the model (i.e. not to get a specific classification model [23]) optimizing the feature selection procedure. A comparative analysis of different machine learning algorithms is out of the scope of the current study (such a comparative analysis between various manifold and machine learning approaches, yet based on global-graph theoretical properties of ICA-based constructed FCN can be found in [27]).

## 5 Conclusions

Using ISOMAP for the construction of brain atlas-based embedded FCN and utilizing different feature selection methods, we found that the most informative features that led to the highest classification rates were the participation coefficient of the right thalamus and the strength of the right lingual gyrus. On the one hand, the thalamus is thought to play a prominent role in the coordination of information as it flows from functional and structural pathways which have been consistently linked to the schizophrenia [74], [75]. Abnormalities in the function of the thalamus have been associated with cognitive deficits and anomalies in sensory experience (e.g. auditory/visual hallucinations) [76]. On the other hand, The lingual gyrus is a main component of the visual cortex that contributes in visual processing/memory [77] and the identification and recognition of words [78]. Many studies have reported abnormalities in the lingual gyrus associated with schizophrenia [79], [80]. Interestingly, recent classification studies involving two independent datasets refer to both of these brain regions as key regions in both characterization of the disease and discrimination between healthy controls and patients with schizophrenia [21], [81]. Other studies have listed these regions as discriminatory in schizophrenia as well [15], [19]. Other important regions found here, include the cuneus and latteral occipital cortex, both parts of the occipital lobe which has been known to be affected as a consequence of the disease [82]. Moreover, our study provides evidence that the use of ISOMAP for the construction of embedded FCN contributes to a more accurate and robust classification plus a finer detection of biomarkers at the ROI level that are more likely to be generalized on other datasets.
